# The addition of PD-1 inhibitor overcame trastuzumab resistance in patients with HER2 positive, PD-L1 negative metastatic gastric cancer: Case report and review of literature

**DOI:** 10.3389/fphar.2024.1447140

**Published:** 2024-10-24

**Authors:** Zhenpeng Wen, Daoli Ye, Qiancheng Hu, Hongfeng Gou

**Affiliations:** ^1^ Department of Medical Oncology, Cancer Center, West China Hospital, Sichuan University, Chengdu, Sichuan, China; ^2^ Gastric Cancer Center, West China Hospital/West China School of Nursing, Sichuan University, Chengdu, Sichuan, China; ^3^ Department of Medical Oncology, Cancer Center, Gastric Cancer Center, West China Hospital, Sichuan University, Chengdu, Sichuan, China

**Keywords:** PD-1 inhibitor, trastuzumab resistance, HER2, PD-L1, gastric cancer

## Abstract

Gastric cancer (GC) is a malignancy with poor prognosis and high heterogeneity. For HER2-positive, PD-L1 negative metastatic GC patients, chemotherapy plus trastuzumab is the first-line therapy. However, such patients soon acquired resistance to treatment, especially to trastuzumab during the treatment. Improving the therapeutic resistance of HER2-positive, PD-L1 negative metastatic GC is still a dilemma. We present the case of a metastatic GC patient with HER2-positive and PD-L1-negative expression who suffered progression after a short remission with trastuzumab plus chemotherapy. The patient exhibited strong heterogeneity in the primary and metastatic lesions. His resistance to trastuzumab was overcome after the addition of a PD-1 inhibitor, after which he received a durable response for more than 8 months. In HER2-positive, PD-L1-negative metastatic GC, the addition of PD-1 inhibitors after first-line chemotherapy and trastuzumab treatment resistance may be an option.

## Introduction

As the fifth most common cancer and the fourth most common cause of death globally, gastric cancer (GC) has a high degree of malignancy and is highly heterogeneous (Sung et al., 2021). Approximately 20% of GC overexpresses human epidermal growth factor receptor 2 (*HER2*), predicting a more aggressive biological behavior and poorer prognosis (Allgayer et al., 2000; Cancer genome atlas research network, 2014). *HER2*, a key driver of tumorigenesis, is a well-established therapeutic target in patients with metastatic GC (Joshi and Badgwell, 2021). Chemotherapy plus trastuzumab is generally the first-line standard of care in patients with HER2-positive metastatic GC, irrespective of programmed death ligand 1 (PD-L1) expression, with a median overall survival of 13.8 months (Bang et al., 2010).

Recently, the third interim analysis of KEYNOTE-811, which included locally advanced or metastatic HER2-positive gastric/GEJ adenocarcinoma, showed that pembrolizumab plus trastuzumab and chemotherapy showed remarkable improvement in complete (16.6% vs. 11.2%) and objective response rates (72.6% vs. 60.1%) compared with trastuzumab and chemotherapy. The progression-free survival benefit with pembrolizumab was significant in patients with tumors with a PD-L1 CPS of 1 or more (Janjigian et al., 2023). Based on this finding, the FDA has approved pembrolizumab plus trastuzumab, fluoropyrimidine and platinum-containing chemotherapy for the first-line treatment of locally advanced or metastatic HER2- and PD-L1-positive (CPS≥1) gastric/GEJ adenocarcinoma. However, for HER2-positive and PD-L1-negative GC patients, the current first-line standard regimen is still chemotherapy combined with trastuzumab. After the first line of trastuzumab combined with chemotherapy, it is still unknown whether the addition of immune checkpoint inhibitors can reverse trastuzumab resistance and provide a survival benefit after trastuzumab resistance.

We here report a case of a 66-year-old male patient with metastatic HER2-positive and PD-L1 negative GC whose trastuzumab resistance was reversed by a PD-1 inhibitor.

## Case presentation

**FIGURE 1:**
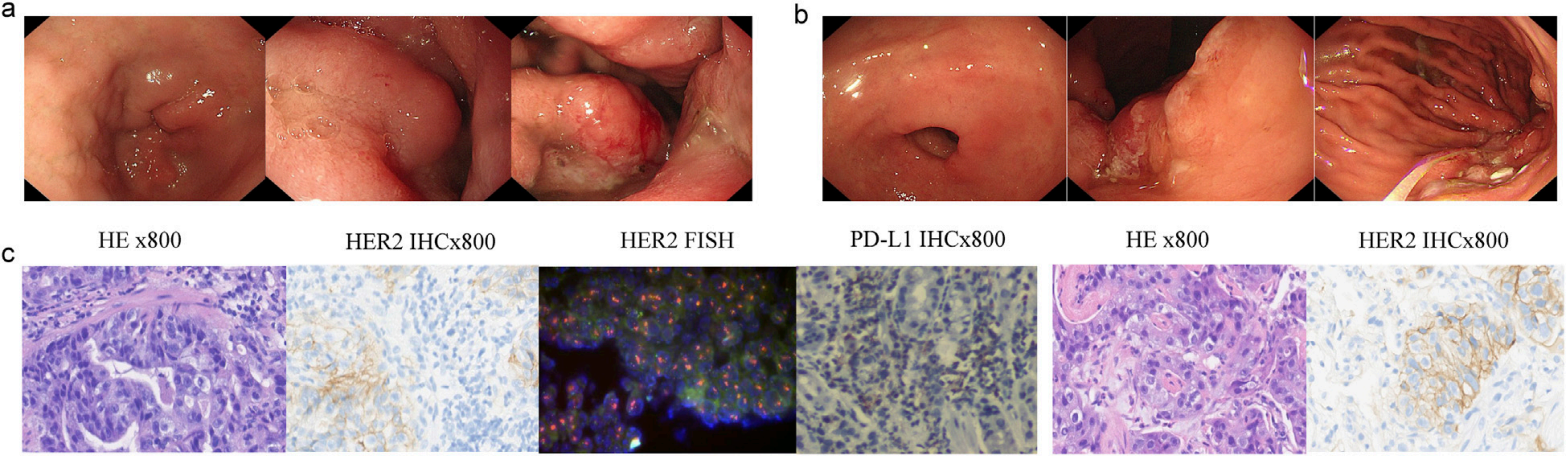
Endoscopy and pathological results patient’s stomach tissue. **(A)** Gastric antrum and body pictures under endoscopy diagnosis. **(B)** Gastric antrum, angle, and body pictures under endoscopy after eight cycles of therapy. **(C)** H&E, HER2, *HER2*, FISH, and PD-L1 staining of the primary gastric cancer tissue; H&E and HER2 staining of the metastatic lung tissue.

**FIGURE 2:**
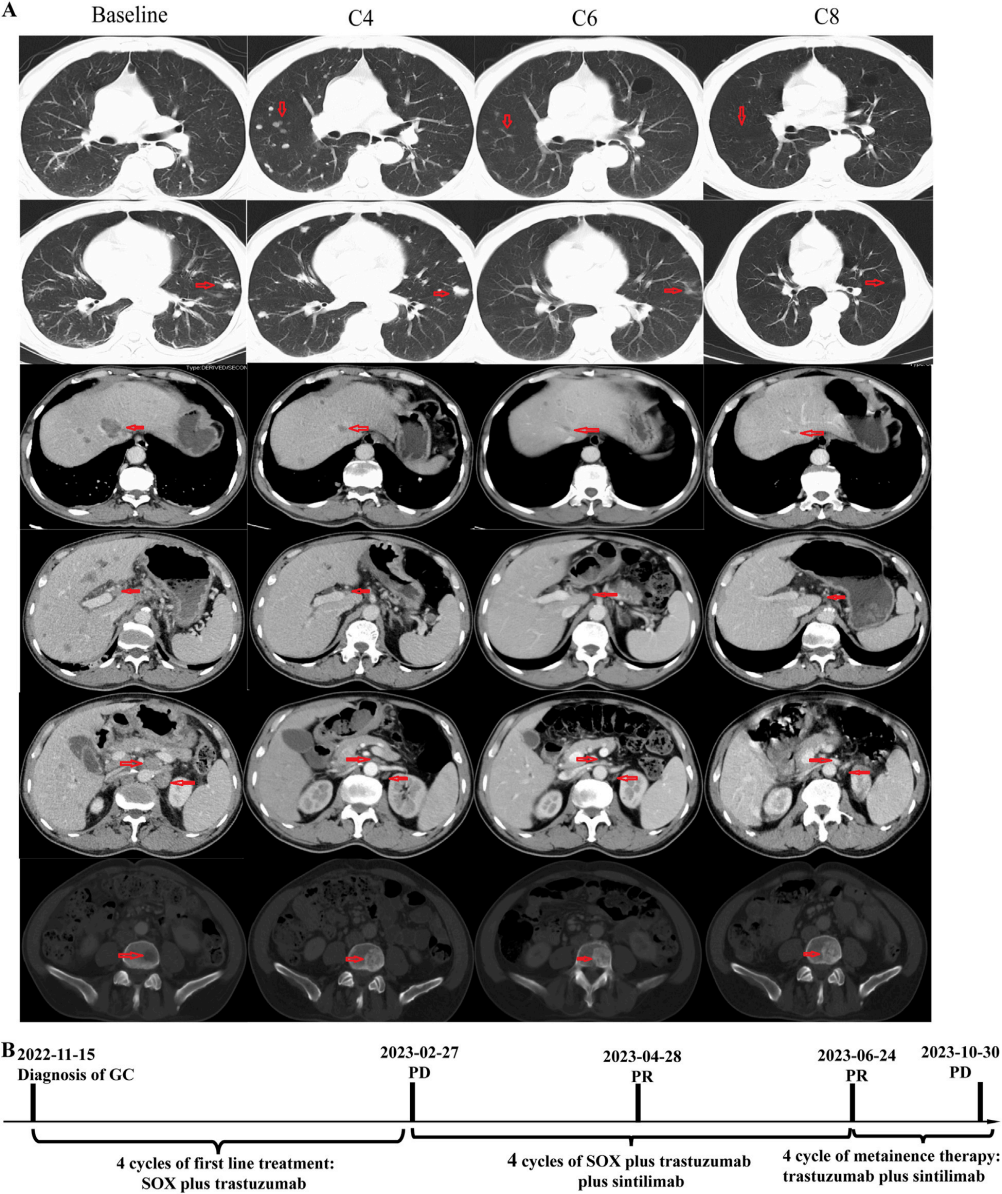
Timeline and representative computed tomography (CT) images. **(A)** CT images showing patient’s baseline disease, progression after four cycles of SOX plus trastuzumab, and response of PD-1 inhibitor plus SOX plus trastuzumab; **(B)** Summary of disease course, treatment procedure, and efficacy evaluation of each treatment procedure.

In November 2022, a 66-year-old Chinese male with a history of hypertension and type II diabetes and no family cancer history or psychosocial disease history was admitted to our hospital after having dull epigastric pain, acid reflux, and melena for 2 months. He received a 6.5-unit red blood cell transfusion because of severe anemia before endoscopy. The latter showed a tumor at his gastric body and swelling of the duodenal mucosa (Figure 1A). The biopsy of his gastric body tissue revealed adenocarcinoma (tubular adenocarcinoma). IHC staining was positive for MSH2, MSH6, MLH1, PMS2, P53 (separated cells, wild type), and HER2(2+) and negative for PD-L1(CPS <1, evaluated by 22C3 antibody) and *EBER*1/2-ISH. *HER2* amplification was validated by FISH (Figure 1C). CT revealed an unevenly enhanced and thickened stomach body with multiple swollen lymph nodes around the small curvature of the stomach, the portal area of the liver, the para-aorta of the abdomen, and the superior mesenteric artery. Sacral bone metastasis from GC was suspected. No apparent peritoneal metastases were present. Chest CT showed multiple micronodules and interstitial pneumonia in both lungs. MRI of the upper abdomen suggested multiple lesions in both liver lobes, considering multiple liver metastases from gastric carcinoma (Figure 2). He was diagnosed with gastric body adenocarcinoma, as well as liver and para-aortic lymph node metastases (cT4bN + M1, stage IVB).

The patient received four cycles of SOX (S-1 orally administered at a dosage of 60 mg twice a day on days 1–14, every 3 weeks; oxaliplatin: intravenously administered at a dose of 220 mg on day 1, every 3 weeks) plus trastuzumab (initial dose: 520 mg intravenously; maintenance dose: 360 mg intravenously, every 3 weeks). A follow-up CT scan in February 2023 revealed disease progression in the lung and bone, but the metastases of the liver and para-aortic lymph nodes showed significant shrinkage. The efficacy evaluation was progressive disease (PD), but there was a marked heterogeneous response in the lung, bone, liver, and para-aortic lymph nodes.

A biopsy of the pulmonary lesion confirmed metastasis from GC. IHC staining was positive for CK7、MLH1、MSH2、MSH6、PMS2、and HER2(2+) and negative for CK20、CDX2、TTF-1、and Napsin A (Figure 1C). A FISH test of *HER2* and a PD-L1 test were not performed due to the patient’s will. The patient received first-line therapy, and multiple new lesions were detected in the lung and bone, but the metastases of the liver and para-aortic lymph nodes were significant shrunken. We negotiated with the patient his next regimen, of either switching to second-line therapy or adding a PD-1 inhibitor to his first-line therapy. After we informed the patient of the possible response and adverse events of PD-1 inhibitor, he chose to add a PD-1 inhibitor, sintilimab, to his previous regimen instead of switching to second-line therapy. Zoledronic acid was used to prevent skeletal-related events in patients with bone metastasis. After two cycles of treatment, efficacy evaluation was PR. The tumor volume continued to shrink during the treatment. Endoscopy showed shrinkage in the GC lesions after eight cycles of treatment (Figure 1B). No immune-related pneumonitis and skeletal-related events were observed. The patient received maintenance therapy of trastuzumab plus sintilimab until CT showed PD on 30 October 2023. The patient had a progression-free survival of more than 8 months until progression. No immune-related or chemotherapy-related adverse events and skeletal-related events were observed.

## Discussion

We reported a patient with metastatic HER2-positive and PD-L1-negative GC whose trastuzumab resistance was reversed by sintilimab. The patient received first-line SOX combined with trastuzumab, and the metastases of the liver and para-aortic lymph nodes were significant shrunken, but multiple new lesions were detected in the lung and bone. Surprisingly, after the addition of a PD-1 inhibitor, all lesions shrank, and the patient achieved sustained PR. Therefore, the combination of PD-1 inhibitors with trastuzumab could be proposed as a possible new strategy for overcoming trastuzumab resistance in patients with HER2-positive, PD-L1-negative metastatic GC.

Trastuzumab, a humanized monoclonal anti-HER2 antibody, combined with chemotherapy significantly improves median overall survival (mOS) in patients with metastatic HER2-positive GC compared to chemotherapy alone (13.8 vs. 11.1 months, HR 0.74; 95% CI 0.60–0.91; *p* = 0.0046). Thus, trastuzumab is approved as the standard frontline of metastatic HER2-positive GC based on the “Trastuzumab for Gastric Cancer” (ToGA) trial (Bang et al., 2010).

Little progress in the first-line treatment of patients with metastatic HER2-positive GC occurred until PD-1 inhibitors emerged. These combined with anti-HER2 therapy was a feasible option for treating patients with HER2-positive GC based on the synergistic mechanism of anti-HER2 therapy and PD-1 inhibitors (Yamashita et al., 2021). In the KEYNOTE-811 trial, the ORR of patients with metastatic HER2 positive GC was improved 12.8% when pembrolizumab was combined with trastuzumab and chemotherapy compared to just trastuzumab and chemotherapy. In May 2021, the FDA approved pembrolizumab in combination with trastuzumab and chemotherapy for the first-line treatment of patients with locally advanced unresectable or metastatic HER2-positive gastric/GEJ adenocarcinoma, regardless of PD-L1 CPS expression, based on the first analysis of KEYNOTE-811 (Janjigian et al., 2021). At the third interim analysis, the HR for OS and PFS in patients (N = 104) with PD-L1 CPS <1 were 1.41 (95% CI 0.90, 2.20) and 1.03 (95% CI 0.65, 1.64), respectively. In patients (N = 594) with PD-L1 CPS≥1, the HR for OS and PFS were 0.81 (95% CI 0.67, 0.98) and 0.70 (95% CI 0.58, 0.85), respectively (Janjigian et al., 2023; US Food and Drug Administration, 2023). Based on the interim analysis, in November 2023 the FDA revised the existing indication of pembrolizumab with trastuzumab and chemotherapy for the first-line treatment of patients (CPS≥1). Recently, results from the final analyses of KEYNOTE-811 showed that in PD-L1-positive patients (CPS ≥1), pembrolizumab combined with trastuzumab and chemotherapy improved PFS (10.9 vs. 7.3 months, HR 0.72; 95 CI% 0.60–0.87) and OS (20.1 vs. 15.7 months, HR 0.79; 95% CI 0.66–0.95) (Janjigian et al., 2024). Data from KEYNOTE-811 have led to European Medicines Agency (EMA) and FDA-approved pembrolizumab combined with trastuzumab and chemotherapy in patients with metastatic HER2- and PD-L1 dual-positive GC. For HER2-positive and PD-L1 negative metastatic GC patients, chemotherapy combined with trastuzumab is still standard treatment. The use of PD-1 inhibitors in second- or third-line patients with metastatic HER2-positive and PD-L1-negative GC needs further investigation.

Nearly half the patients with metastatic HER2-positive GC experience disease progression or death after trastuzumab treatment, and the mechanisms of drug resistance are relatively complex and uncertain (Bang et al., 2010; Valabrega et al., 2007). Second-line standard therapy, such as ramucirumab and/or paclitaxel, might be offered to patients with progression disease after first-line treatments. In addition, continued anti-HER2 therapy is an option. The HER2-targeted antibody–drug conjugates, such as trastuzumab deruxtecan (T-DXd/DS-8201) and disitamab vedotin (RC48), have shown clinical benefit after trastuzumab resistance. The ORR of T-DXd as second-line therapy in patients with HER2-positive advanced gastric/GEJ adenocarcinoma was 42% (95% CI: 30.8–53.4). Third-line or subsequent treatment of T-DXd for HER2-positive GC patients showed longer OS than chemotherapy (median, 12.5 vs. 8.4 months; HR for death, 0.59; 95% CI: 0.39 to 0.88; *P* = 0.01. The ORR was 24.8% (95% CI: 17.5%–33.3%) (Shitara et al., 2020). The median PFS and OS were 4.1 months (95% CI: 3.7–4.9 months) and 7.9 months (95% CI: 6.7–9.9 months) for RC48 as third-line or subsequent treatment for HER2-positive advanced gastric/GEJ adenocarcinoma (Peng et al., 2021). Both the FDA and EMA have approved T-DXd in adult patients with locally advanced or metastatic HER2-positive gastric/GEJ adenocarcinoma who had received a trastuzumab-based regimen (US Food and Drug Administration, 2021; European Medicines Agency, 2021). Research has also explored the continuation of trastuzumab with new chemotherapy regimens beyond progression disease. Although continuing treatment of trastuzumab combined with new chemotherapy regimens beyond first-line therapy progression have shown possibly effective in some retrospective studies (Palle et al., 2017; Li et al., 2016; Narita et al., 2017), a randomized prospective T-ACT study showed that the strategy had no benefit (Makiyama et al., 2020). A new combination of trastuzumab beyond progression was further explored. A phase Ib/II open-label single-arm trial of trastuzumab in combination with ramucirumab and paclitaxel has recently reported very promising gains in median progression-free (7.1 months, 95% CI: 4.8–9.4) and overall survival (13.6 months, 95% CI: 9.4–17.7) in patients with previously treated metastatic HER2-positive GC (Kim et al., 2023).

Here, we report a case who experienced progression disease after first-line trastuzumab plus chemotherapy. At that point, T-DXd was not feasible in China, and RC48 was approved for the third-line treatment of HER2-positive gastric or GEJ adenocarcinoma. The FDA accelerated the approval for pembrolizumab in combination with trastuzumab and chemotherapy in HER2-positive gastric or GEJ adenocarcinoma based on the ORR in the first analysis of KEYNOTE-811. In this case, despite progressive lung and bone disease, liver metastases, and para-aortic lymph-node metastases shrank markedly. We thus negotiated with the patient that the addition of sintilimab to his first-line therapy was an option. Partial response was seen for more than 8 months. In the patient, it was observed that PD-1 inhibitor could overcome trastuzumab resistance in PD-L1 negative tumors. What then are the potential mechanisms? PD-L1 upregulation was thought to be the mechanism of trastuzumab resistance. Yamashita et al. (2021) observed PD-L1 upregulation by trastuzumab in GC cells. In breast cancer cells, trastuzumab stimulated INF-gamma secretion to upregulate PD-L1 (Chaganty et al., 2018). In *in vivo* models, anit-PD-1 or -CD137 could improve the therapeutic activity of anti-HER2 (Stagg et al., 2011). Pembrolizumab in combination with trastuzumab had shown activity in patients with trastuzumab-resistant, advanced HER2-positive breast cancer (Loi et al., 2019). In a single-arm phase 1b-2 trial which explored margetuximab and pembrolizumab combination treatment in patients with HER2-positive gastric/GEJ adenocarcinoma, the synergistic antitumor activity of anti-HER2 agent (margetuximab) with anti-PD-1 checkpoint blockade (pembrolizumab) was observed in patients of previously treated HER2 positive gastric/GEJ adenocarcinoma (Catenacci et al., 2020). Apart from this, there is also trial (NCT05270889) recruiting previously treated HER2-positive advanced gastric/GEJ adenocarcinoma patients to assess the safety and clinical efficacy of this combined chemotherapy-free regimen (zanidatamab and tislelizumab) in second-line therapy. Other contributing factors could be the dynamic of PD-L1 expression and the heterogeneity of PD-L1 expression in tumor tissues (Kerr and Hirsch, 2016). More studies are warranted to validate this result, especially for patients with metastatic HER2-positive and PD-L1-negative GC.

The heterogeneity of HER2 is a pivotal reason why anti-HER2 therapies fail in patients with metastatic HER2-positive GC (Roviello et al., 2021). Temporal and spatial heterogeneity of HER2 might be important in predicting response to anti-HER2 therapies (Marusyk et al., 2020; Wolff et al., 2018; Lee et al., 2013; Hofmann et al., 2008; Rüschoff et al., 2012). Loss of HER2 expression was detected in 34.8% of patients after first-line anti-HER2 therapies (Kim et al., 2023). Hence, it is necessary to monitor the dynamic of HER2 expression for subsequent treatments to be developed. To test whether there was a spatial heterogeneity of HER2 in our case, a CT-guided needle biopsy of the left lung was performed, with the HER2 expression still a 2+ score by IHC. Thus, it was postulated that the patient might necessarily gain a clinically significant benefit from the continuation of trastuzumab. Lastly, our clinical evidence substantiated this speculation in our case.

We here reported a rare case of PD-1 inhibitors overcoming trastuzumab resistance in patients with metastatic HER2-positive and PD-L1-negative GC. For this specific type of patient, continued use of anti-HER2 drugs combined with the immune checkpoint blockade may be a new regimen. More studies are encouraged to investigate the underlying mechanisms that trastuzumab resistance can be overcome by PD-1 inhibitors.

## Data Availability

The raw data supporting the conclusions of this article will be made available by the authors, without undue reservation.

## References

[B1] Allgayer H. Babic R. Gruetzner K. U. Tarabichi A. Schildberg F. W. Heiss M. M. (2000). c-erbB-2 is of independent prognostic relevance in gastric cancer and is associated with the expression of tumor-associated protease systems. J. Clin. Oncol. 18, 2201–2209. 10.1200/JCO.2000.18.11.2201 10829039

[B2] Bang Y.-J. Van Cutsem E. Feyereislova A. Chung H. C. Shen L. Sawaki A. (2010). Trastuzumab in combination with chemotherapy versus chemotherapy alone for treatment of HER2-positive advanced gastric or gastro-oesophageal junction cancer (ToGA): a phase 3, open-label, randomised controlled trial. Lancet 376, 687–697. 10.1016/s0140-6736(10)61121-x 20728210

[B3] Cancer genome atlas research network (2014). Comprehensive molecular characterization of gastric adenocarcinoma. Nature 513, 202–209. 10.1038/nature13480 25079317 PMC4170219

[B4] Catenacci D. V. T. Kang Y.-K. Park H. Uronis H. E. Lee K.-W. Ng M. C. H. (2020). Margetuximab plus pembrolizumab in patients with previously treated, HER2-positive gastro-oesophageal adenocarcinoma (CP-MGAH22-05): a single-arm, phase 1b-2 trial. Lancet Oncol. 21, 1066–1076. 10.1016/S1470-2045(20)30326-0 32653053

[B5] Chaganty B. K. R. Qiu S. Gest A. Lu Y. Ivan C. Calin G. A. (2018). Trastuzumab upregulates PD-L1 as a potential mechanism of trastuzumab resistance through engagement of immune effector cells and stimulation of IFNγ secretion. Cancer Lett. 430, 47–56. 10.1016/j.canlet.2018.05.009 29746929 PMC6004098

[B6] European Medicines Agency (2021). EMA approves Enhertu for HER2-positive advanced gastric cancer or gastro-oesophageal junction cancer patients who have previously received trastuzumab treatment, south Amsterdam. Available at: https://www.ema.europa.eu/en/medicines/human/EPAR/enhertu.

[B7] Hofmann M. Stoss O. Shi D. Büttner R. van de Vijver M. Kim W. (2008). Assessment of a HER2 scoring system for gastric cancer: results from a validation study. Histopathology 52, 797–805. 10.1111/j.1365-2559.2008.03028.x 18422971

[B8] Janjigian Y. Y. Kawazoe A. Bai Y. Xu J. Lonardi S. Metges J. P. (2023). Pembrolizumab plus trastuzumab and chemotherapy for HER2-positive gastric or gastro-oesophageal junction adenocarcinoma: interim analyses from the phase 3 KEYNOTE-811 randomised placebo-controlled trial. Lancet 402, 2197–2208. 10.1016/s0140-6736(23)02033-0 37871604

[B9] Janjigian Y. Y. Kawazoe A. Bai Y. Xu J. Lonardi S. Metges J. P. (2024). 1400O Final overall survival for the phase III, KEYNOTE-811 study of pembrolizumab plus trastuzumab and chemotherapy for HER2+ advanced, unresectable or metastatic G/GEJ adenocarcinoma. Ann. Oncol. 35, S877–S878. 10.1016/j.annonc.2024.08.1466

[B10] Janjigian Y. Y. Kawazoe A. Yañez P. Li N. Lonardi S. Kolesnik O. (2021). The KEYNOTE-811 trial of dual PD-1 and HER2 blockade in HER2-positive gastric cancer. Nature 600, 727–730. 10.1038/s41586-021-04161-3 34912120 PMC8959470

[B11] Joshi S. S. Badgwell B. D. (2021). Current treatment and recent progress in gastric cancer. CA Cancer J. Clin. 71, 264–279. 10.3322/caac.21657 33592120 PMC9927927

[B12] Kerr K. M. Hirsch F. R. (2016). Programmed death ligand-1 immunohistochemistry: friend or foe? Arch. Pathol. Lab. Med. 140, 326–331. 10.5858/arpa.2015-0522-SA 26756647

[B13] Kim C. G. Jung M. Kim H. S. Lee C.-K. Jeung H.-C. Koo D.-H. (2023). Trastuzumab combined with ramucirumab and paclitaxel in patients with previously treated human epidermal growth factor receptor 2-positive advanced gastric or gastroesophageal junction cancer. J. Clin. Oncol. 41, 4394–4405. 10.1200/jco.22.02122 37364218

[B14] Lee H. E. Park K. U. Yoo S. B. Nam S. K. Park D. J. Kim H.-H. (2013). Clinical significance of intratumoral HER2 heterogeneity in gastric cancer. Eur. J. Cancer 49, 1448–1457. 10.1016/j.ejca.2012.10.018 23146959

[B15] Li Q. Jiang H. Li H. Xu R. Shen L. Yu Y. (2016). Efficacy of trastuzumab beyond progression in HER2 positive advanced gastric cancer: a multicenter prospective observational cohort study. Oncotarget 7, 50656–50665. 10.18632/oncotarget.10456 27409420 PMC5226611

[B16] Loi S. Giobbie-Hurder A. Gombos A. Bachelot T. Hui R. Curigliano G. (2019). Pembrolizumab plus trastuzumab in trastuzumab-resistant, advanced, HER2-positive breast cancer (PANACEA): a single-arm, multicentre, phase 1b-2 trial. Lancet Oncol. 20, 371–382. 10.1016/s1470-2045(18)30812-x 30765258

[B17] Makiyama A. Sukawa Y. Kashiwada T. Kawada J. Hosokawa A. Horie Y. (2020). Randomized, phase II study of trastuzumab beyond progression in patients with HER2-positive advanced gastric or gastroesophageal junction cancer: WJOG7112G (T-ACT study). J. Clin. Oncol. 38, 1919–1927. 10.1200/JCO.19.03077 32208960

[B18] Marusyk A. Janiszewska M. Polyak K. (2020). Intratumor heterogeneity: the rosetta stone of therapy resistance. Cancer Cell 37, 471–484. 10.1016/j.ccell.2020.03.007 32289271 PMC7181408

[B19] Narita Y. Kadowaki S. Masuishi T. Taniguchi H. Takahari D. Ura T. (2017). Correlation between human epidermal growth factor receptor 2 expression level and efficacy of trastuzumab beyond progression in metastatic gastric cancer. Oncol. Lett. 14, 2545–2551. 10.3892/ol.2017.6409 28781693 PMC5530221

[B20] Palle J. Tougeron D. Pozet A. Soularue E. Artru P. Leroy F. (2017). Trastuzumab beyond progression in patients with HER2-positive advanced gastric adenocarcinoma: a multicenter AGEO study. Oncotarget 8, 101383–101393. 10.18632/oncotarget.20711 29254172 PMC5731882

[B21] Peng Z. Liu T. Wei J. Wang A. He Y. Yang L. (2021). Efficacy and safety of a novel anti-HER2 therapeutic antibody RC48 in patients with HER2-overexpressing, locally advanced or metastatic gastric or gastroesophageal junction cancer: a single-arm phase II study. Cancer Commun. (Lond) 41, 1173–1182. 10.1002/cac2.12214 34665942 PMC8626607

[B22] Roviello G. Aprile G. D'Angelo A. Iannone L. F. Roviello F. Polom K. (2021). Human epidermal growth factor receptor 2 (HER2) in advanced gastric cancer: where do we stand? Gastric Cancer 24, 765–779. 10.1007/s10120-021-01182-9 33742317

[B23] Rüschoff J. Hanna W. Bilous M. Hofmann M. Osamura R. Y. Penault-Llorca F. (2012). HER2 testing in gastric cancer: a practical approach. Mod. Pathol. 25, 637–650. 10.1038/modpathol.2011.198 22222640

[B24] Shitara K. Bang Y.-J. Iwasa S. Sugimoto N. Ryu M.-H. Sakai D. (2020). Trastuzumab deruxtecan in previously treated HER2-positive gastric cancer. N. Engl. J. Med. 382, 2419–2430. 10.1056/NEJMoa2004413 32469182

[B25] Stagg J. Loi S. Divisekera U. Ngiow S. F. Duret H. Yagita H. (2011). Anti-ErbB-2 mAb therapy requires type I and II interferons and synergizes with anti-PD-1 or anti-CD137 mAb therapy. Proc. Natl. Acad. Sci. U. S. A. 108, 7142–7147. 10.1073/pnas.1016569108 21482773 PMC3084100

[B26] Sung H. Ferlay J. Siegel R. L. Laversanne M. Soerjomataram I. Jemal A. (2021). Global cancer statistics 2020: GLOBOCAN estimates of incidence and mortality worldwide for 36 cancers in 185 countries. CA Cancer J. Clin. 71, 209–249. 10.3322/caac.21660 33538338

[B27] US Food and Drug Administration (2021). FDA approves fam-trastuzumab deruxtecan-nxki for HER2-positive gastric adenocarcinomas. Available at: https://www.fda.gov/drugs/resources-information-approved-drugs/fda-approves-fam-trastuzumab-deruxtecan-nxki-her2-positive-gastric-adenocarcinomas.

[B28] US Food and Drug Administration (2023). FDA amends pembrolizumab’s gastric cancer indication. Available at: https://www.fda.gov/drugs/resources-information-approved-drugs/fda-amends-pembrolizumabs-gastric-cancer-indication.

[B29] Valabrega G. Montemurro F. Aglietta M. (2007). Trastuzumab: mechanism of action, resistance and future perspectives in HER2-overexpressing breast cancer. Ann. Oncol. 18, 977–984. 10.1093/annonc/mdl475 17229773

[B30] Wolff A. C. Hammond M. E. H. Allison K. H. Harvey B. E. Mangu P. B. Bartlett J. M. S. (2018). Human epidermal growth factor receptor 2 testing in breast cancer: American society of clinical oncology/college of American pathologists clinical practice guideline focused update. Arch. Pathol. Lab. Med. 142, 1364–1382. 10.5858/arpa.2018-0902-SA 29846104

[B31] Yamashita K. Iwatsuki M. Yasuda-Yoshihara N. Morinaga T. Nakao Y. Harada K. (2021). Trastuzumab upregulates programmed death ligand-1 expression through interaction with NK cells in gastric cancer. Br. J. Cancer 124, 595–603. 10.1038/s41416-020-01138-3 33100329 PMC7851117

